# Evaluation of real-time PCR for diagnosis of *Bordetella pertussis *infection

**DOI:** 10.1186/1471-2334-6-62

**Published:** 2006-03-23

**Authors:** Laina Knorr, Julie D Fox, Peter AG Tilley, Jasmine Ahmed-Bentley

**Affiliations:** 1 Provincial Laboratory for Public Health, Calgary, Alberta, Canada; 2 Department of Medical Microbiology, University of Alberta, Edmonton, Alberta, Canada; 3 Department of Microbiology and Infectious Diseases, University of Calgary, Alberta, Canada

## Abstract

**Background:**

Nucleic acid amplification of the *IS481 *region by PCR is more sensitive than culture for detection and diagnosis of *Bordetella pertussis *but the assay has known cross-reactivity for *Bordetella holmesii *and its use as a routine diagnostic assay has not been widely evaluated.

**Methods:**

The objectives of this study were: 1) to assess the diagnostic utility of real-time *IS481 *PCR by comparison of results with culture and direct fluorescent antigen (DFA) testing for *B. pertussis*, 2) to employ a PCR assay designed against a different insertion sequence (*IS1001) *to assess the incidence of *B. holmesii *in symptomatic individuals and 3) to design and evaluate a new PCR-based assay which could be used for *B. pertussis *confirmation. A total of 808 nasopharyngeal specimens were included in the study the majority of which were submitted in charcoal transport medium (88%) with the rest submitted in Regan-Lowe medium.

**Results:**

Concordant results for PCR, DFA and culture were obtained for 21 *B. pertussis *positive and 729 *B. pertussis *negative specimens. DFA was prone to false positive and negative reactions when compared with both PCR and culture. The *IS481 *PCR identified 28 positive results for specimens that were DFA and culture negative. A novel real-time PCR targeting the *B. pertussis *toxin promoter was found to be specific and useful for confirming the majority of *IS481 *positive specimens as *B. pertussis*. *B. holmesii *was not detected in any of the submitted samples.

**Conclusion:**

The potential pick up of *B. holmesii *by the *IS481 *PCR had minimal diagnostic relevance in the Alberta population during the time period of our study. The *IS481 *PCR assay is now used in our laboratory routinely for front-line screening of samples for *B. pertussis *with associated enhancement in diagnostic sensitivity compared with DFA and culture. Retrospectively, patients' samples are batched and tested by the *IS1001 *MB and TPR assays for research purposes and to ensure there is no change in *B. holmesii *incidence in the population.

## Background

The etiologic agent of whooping cough is *Bordetella pertussis*. Diagnosis at an early stage of illness is desired to institute therapy and prevent transmission. Pertussis-like respiratory illness can also be caused by other *Bordetella *species including *parapertussis *[1], *bronchiseptica *[2] and *holmesii *[3,4]. Currently available *B. pertussis *diagnostic methods include culture, direct fluorescent antigen (DFA) tests, serology and nucleic acid amplification assays such as the polymerase chain reaction (PCR). The first publication of *B. pertussis *PCR was in 1989 [5]. Since then numerous protocols have been reported targeting different bacterial genes. *B. pertussis *PCR can be applied directly to nasopharyngeal specimens, is more sensitive than culture, can detect nonviable organisms and results using this method can be obtained rapidly. Thus, this diagnostic approach has the potential to improve our understanding of *B. pertussis *transmission and epidemiology, to allow us to measure accurately the impact on the community, as well as enhance studies of vaccine efficacy.

*B. pertussis *carries 50–100 copies of *IS481 *and therefore this should be a good target for development of a sensitive diagnostic assay when compared with other single-copy regions or genes. Indeed, *IS481*-based PCR has been shown to be more sensitive than one designed against the toxin promoter [6]. It has been shown that PCR assays targeting *IS481 *PCR are highly specific for *B. pertussis *and *B. holmesii *[7] and, in a real-time format, this approach enhances diagnostic utility above other methods. However, the potential detection of *B. holmesii *in cases of a pertussis-like illness could compromise appropriate management and compromise studies of vaccine efficacy [7].

In this study we aimed to assess the diagnostic utility of an *IS481 *real-time PCR assay [7], compare the PCR results to culture and DFA, assess the incidence of *B. holmesii *in the test population and develop and apply a novel real-time PCR assay against the toxin promoter region for specific detection (and thus confirmation) of *B. pertussis *infection.

## Methods

### Bacterial strains

All bacteria used for the study were from standard cultures grown at 35°C in ambient atmosphere on Regan-Lowe media (Daylnn Biologicals Inc., Calgary, AB). *B. pertussis*, *B. holmesii*, *B. bronchiseptica *and *B. parapertussis *cultures were suspended in sterile saline at a concentration equivalent to 10^8 ^cfu/ml, based on McFarland turbidimetric standards.

### Processing of specimens

Consecutive nasopharyngeal (NP) specimens received during 2003 (n = 204) and 2004 (n = 604) for *B. pertussis *DFA and culture testing were used for the PCR validation study. These specimens were collected and submitted from clinics across Southern Alberta to the Provincial Laboratory for Public Health (ProvLab; Calgary, AB). Clinical data were not collected. Dacron NP swabs (Oxoid Inc., Nepean, ON) were received in either charcoal (Copan Diagnostics Inc., Corona, CA; 88 % of the specimens) or Regan-Lowe (Daylnn Biologicals Inc., Calgary, AB; 12 % of the specimens) transport media. The Regan Lowe medium contained cephalexin which would inhibit *B. holmesii *and prevent its identification by culture. Swabs were placed into 50 μl of 1 % (w/v) casamino acid (Difco, Becton Dickison, Sparks, MD) diluted in PBS. The wire from the NP swab was cut and then, after brief vortexing to remove cellular material into the fluid, the swab was removed. The casamino acid volume was split for bacteriology (DFA/culture) and molecular testing (PCR). If a DFA slide was submitted in addition to the NP swab 20 μl of the swab/casamino acid was used each for culture and PCR. If a DFA slide was not received with the patient swab, the casamino acid was split and 10 μl of the swab/casamino acid was used each for DFA, culture and PCR. Whether a slide for DFA was submitted or not, the volume of sample used for analysis by PCR or culture was always the same.

### DFA

DFA testing was performed using a commercial monoclonal antibody reagent, according to the manufacturer's instructions (Accu-MAb™ Plus, Altachem Pharma Inc., Edmonton, AB).

### Culture

For culture, the 10 or 20 μl of patient specimen was made up to 200 μl with 1% casamino acid and split equally between two Regan-Lowe plates, one with and one without cephalexin (Daylnn). Cultures were held for 7 days and inspected daily. Opalescent colonies were gram stained. If gram negative rods or coccobacilli were seen a suspension of colony was prepared for identification using the fluorescein conjugated monoclonal antibody above.

### DNA extraction

Nucleic acid was extracted from bacterial suspensions and clinical specimens using the automated MagNa Pure LC™ and DNA Isolation Kit I (Roche Diagnostics, Laval, QC). The 10 or 20 μl of patient specimen was made up to 200 μl with physiological saline prior to extraction and elution was into 100 μl volume. A negative control of 200 μl saline was included. Ten μl of a *B. pertussis *control (ATCC 9797 at 10^6 ^cfu/ml) was made up to 200 μl with saline and used as a positive extraction control.

### Primers and probes

The primers used in all assays were prepared by the University of Calgary Core DNA Synthesis facility. The probes used in all assays were prepared by Applied BioSystems (Foster City, CA).

### *IS481 *Hybridization probe assay

Primers and probes used in the *IS481 *hybridization probe (*IS481 HP*) assay are as described previously [7]. Real-time PCR was performed in a 20 μl reaction mixture with final concentrations of 1X LightCycler™ FastStart DNA master hybridization probes mix (Roche Diagnostics), 3 mM MgCl_2_, 0.5 μM of each primer, 0.2 μM of each hybridization probe and 5 μl of extracted template DNA. The amplification profile consisted of activation of enzyme at 95°C for 10 min followed by 50 cycles consisting of heating at 20°C/s to 95°C with a 10s hold, cooling at 20°C/s to 50°C with a 10s hold and heating at 20°C/s to 72°C with a 10s hold. Single fluorescent readings were taken at the annealing temperature (55 °C, once per cycle). Amplification, detection and data analysis were performed on the LightCycler™ 2.0 with software version 4.0 (Roche Diagnostics).

### *IS481 *and *IS1001 *molecular beacon assays

Primers and molecular beacons used in the *IS481 *and *IS1001 *molecular beacon (MB) assays are previously described [8]. The *IS481 *MB assay targets the *IS481 *region for detection of *B. pertussis *and *B. holmesii *and the *IS1001 *MB assay targets the *IS1001 *region for detection of *B. parapertussis *and *B. holmesii*.

These assays were originally designed for the iCycler (BioRad, Hercules, CA), but the assays were adapted for use on the LightCycler™ for the purposes of this study. Initial optimization involved testing a range of MgCl_2 _concentrations and varying annealing temperatures and cycling times in the amplification profile, using serial dilutions of purified *B. pertussis *(ATCC 9797), *B. holmesii *(ATCC 51541) and *B. parapertussis *(ATCC 1533) DNA.

The optimized *IS481 *MB assay was performed in a 20 μl of reaction mixture with final concentrations of 1X LightCycler™ FastStart DNA master hybridization probes mix, 3 mM MgCl_2_, 0.1 μM of each primer, 0.3 μM of the *IS481 *molecular beacon and 5 μl of extracted template DNA. The optimized *IS1001 *MB assay was performed in 20 μl of reaction mixture with final concentrations of 1X LightCycler™ FastStart DNA master hybridization probes mix, 4.5 mM MgCl_2_, 0.2 μM of each primer, 0.4 μM of the *IS1001 *molecular beacon and 5 μl of extracted template DNA. The amplification profile for both assays consisted of activation of enzyme at 95°C for 10 min followed by 50 cycles consisting of heating at 20°C/s to 95°C with a 5 s hold, cooling at 20°C/s to 55°C with a 20 s hold, and heating at 10°C/s to 72°C with a 15 s hold. Single fluorescent readings were taken at the annealing temperature (55°C, once per cycle). Amplification, detection and data analysis were performed on the LightCycler™ 2.0 with software version 4.0.

### Development of a novel toxin promoter region (TPR) hydrolysis probe assay

**Figure 1:**
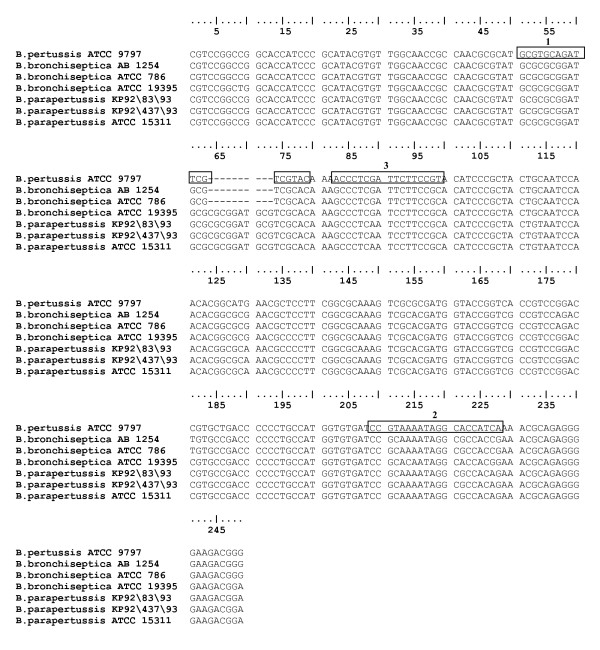
**Alignment based on *B. pertussis *ATCC 9797 with primers and probe for real-time PCR indicated. **The selected forward primer is labeled box 1 (base pairs 51–69) the reverse primer as box 2 (base pairs 218–199) and the probe by box 3 (base pairs 73–89). GenBank accession numbers for each isolate as listed in order above are AF157344, AF157363, AF157362, AF157364, AF157347, AF157357, AF157361, respectively.

Selected sequences of the toxin promoter gene of *B. pertussis *with 100% homology in a 239 bp region were obtained from GenBank (accession numbers AF157344, AF157344, AF157332, AF157342, AF157341, AF157340, AF157337), aligned and a consensus sequence determined (BioEdit, version 7.0.1, Isis Pharmaceuticals Inc., Carlsbad, CA). This consensus was then aligned with 3 different strains of *B. parapertussis *and *B. bronchiseptica *to determine significant polymorphic regions (Figure 1). Primers and a probe were designed from the *B. pertussis *consensus sequence using PrimerExpress software (Applied BioSystems, Foster City, CA) with some adaptations to incorporate the polymorphic regions. The probe was designed with a FAM fluorescent reporter on the 5' end and non-fluorescent black hole quencher (BNFQ) at the 3' end. PrimerExpress was used to ensure minimal self-complementary and secondary structures for primers and probe. A BLAST search of available sequences in the GenBank database was performed to verify the specificity of the primers and probe. Several primer pairs were tested by conventional PCR to determine the combination of forward and reverse primers that performed optimally. The primer pair that was finally chosen for conventional and real-time PCR amplified a 167 bp region. Sequences of optimized primers and hydrolysis probe were as follows:

Forward TPR primer 5'GCGTGCAGATTCGTCGTAC3'

Reverse TPR primer 5'TGATGGTGCCTATTTTACGG3'

Hydrolysis (TaqMan) TPR probe 5'FAM-ACCCTCGATTCTTCCGT-BNFQ3'

Conventional PCR was performed in 50 μl of reaction mixture with final concentrations of 1X PCR buffer (Invitrogen Canada Inc., Burlington, ON), 2 mM MgCl_2_, 0.1 mM of each dNTP (Invitrogen), 0.4 μM of each primer, 1.25 U of DNA *Taq *polymerase (Invitrogen) and 5 μl of extracted template DNA. The amplification profile consisted of an initial incubation of 3 min at 95°C followed by 40 cycles of 30 s at 95°C, 30 s at 55°C and 30 s at 72°C, with a final 10 min hold at 72°C. The 167 bp *B. pertussis *product was separated by agarose gel electrophoresis (2 % agarose containing 0.5 μg/ml ethidium bromide in 0.5 × TBE buffer). Amplified products were viewed and photographed under UV light.

The real-time TPR assay was performed on the LightCycler™ with a variety of MgCl_2_, primers and probe concentrations. The optimized reaction was undertaken in a 20 μl reaction mixture with final concentrations of 1X LightCycler FastStart DNA master hybridization probes mix, 3 mM MgCl_2_, 0.5 μM each primer, 0.1 μM probe, and 5 μl of extracted template DNA. The amplification profile consisted of activation of enzyme at 95°C for 10 min followed by 50 cycles consisting of heating at 20°C/s to 95°C with a 5 s hold, cooling at 20°C/s to 55°C with a 20 s hold and heating at 10°C/s to 72°C with a 15 s hold. Single fluorescent readings were taken at the annealing temperature (55°C, once per cycle). Amplification, detection and data analysis were performed on the LightCycler™ 2.0 with software version 4.0.

### Reporting of results and data analysis

For this laboratory based study a positive result for any PCR or culture-based assay was considered a "true positive" for analysis. Only conventional DFA and culture were reported to submitting physicians during this study period.

## Results

### Analytical sensitivity and specificity of the *IS481 *HP assay

**Figure 2:**
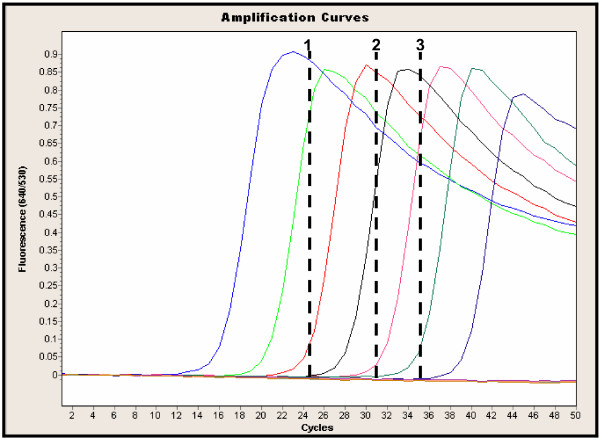
**LightCycler™ amplification profile of the *IS481 *HP probe assay with a dilution series of *B. pertussis ***ATCC 9797 strain of *B. pertussis *was utilized (10^6 ^cfu/ml to 10^-3 ^cfu/ml). Analytical sensitivity of this assay is 10 cfu/ml (0.1 cfu input). The dashed lines correspond to the average Ct value where conventional results are: 1 = DFA positive and culture positive, 2 = DFA negative, culture positive, 3 = DFA negative, culture negative. HP = hybridization probe, Ct = crossing threshold, DFA = direct fluorescent antigen

The *IS481 *HP assay was performed on a serial dilution of purified DNA of *B. pertussis *ATCC 9797 to determine the limit of detection for this assay. This assay gave a good positive signal from 10 cfu/ml stock reproducibly (equivalent to 0.1 cfu input into the assay; Figure 2). The 1 cfu/ml stock (input 0.01 cfu/ml) gave a positive result in approximately 20% of tests. The same positive control stock preparation was used as the processed positive control in the validation study, at 5000 cfu/ml and had an average Ct value of 26.0 (± 2.0) cycles. This corresponds to an inter-assay coefficient of variation of approximately 8 % for this control. As expected, this assay picked up *B. holmesii *as well as *B. pertussis *but not *B. parapertussis *[7]. Further assessment of specificity for this assay was not undertaken but has been evaluated elsewhere [7].

### Comparison of the *IS481 *HP assay with conventional methods

**Figure 3:**
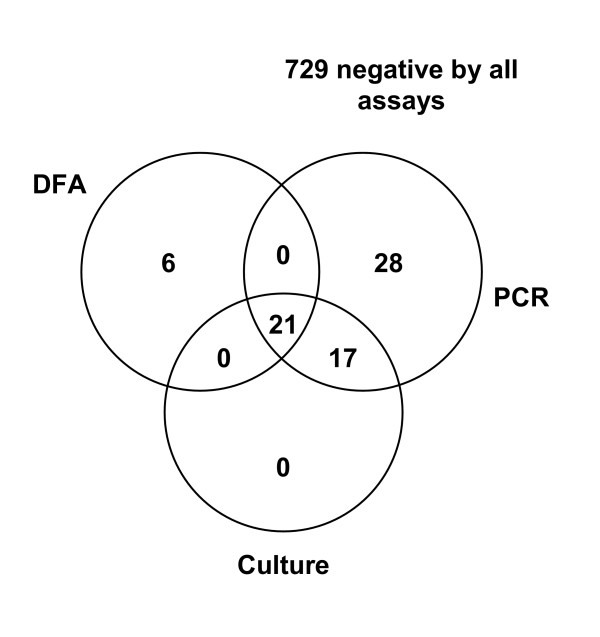
**Correlation of positive results for specimens tested by DFA, culture and the *IS481 *HP PCR assay for *B. pertussis*w **HP = hybridization probe, DFA = direct fluorescent antigen Seven specimens were overgrown in culture and thus have not been included in the Figure (see text for details).

A total of 808 specimens were tested by PCR for *B. pertussis *during this study between March 2003 and April 2004 (204 specimens from 2003 and 604 from 2004). Figure 3 summarizes the results and Table 1 shows the average crossing threshold (Ct) and standard deviation (SD) for positive specimens for each combination of results.

**Table 1:** Comparison of positive *B. pertussis *patient specimens by real-time RT-PCR assays Average crossing threshold (Ct) values and standard deviations (SD) for each combination of PCR and conventional results are shown. Samples were selected based on results for DFA/culture *IS481 *HP PCR.

**Result combination**	***IS481 *HP Assay**			*IS481 *MB Assay*			TPR Assay*		
	
	**Positive/tested (%)**	**Average Ct of positives**	**SD of positives**	**Positive/tested (%)**	**Average Ct of positives**	**SD of positives**	**Positive/tested (%)**	**Average Ct of positives**	**SD of positives**
DFA negative Culture negative Screen PCR (*IS481*HP) positive	28/28 (100.0)	35.7	3.9	26/28 (92.9)	40.4	3.2	15/28 (53.6)	40.1	2.1
DFA negative Culture positive Screen PCR (*IS481*HP) positive	17/17 (100.0)	31.6	5.4	17/17 (100.0)	36.5	5.7	15/17 (88.2)	37.7	5.0
DFA positive Culture positive Screen PCR (*IS481*HP) positive	21/21 (100.0)	24.9	3.8	21/21 (100.0)	30.1	3.9	21/21 (100.0)	32.3	3.4

*In cases where cycle results were >45, 45 was used to calculate average Ct values and SD. HP = hybridization probe, MB = molecular beacon, TPR = toxin promoter region, DFA = direct fluorescent antigen.

Of the 808 samples tested, 729 were negative by all 3 methods. Nine contained detectable *IS481 *target DNA in a single run but results were not reproducible and finally were reported as not containing detectable *B. pertussis*. These specimens were weakly positive [average Ct of 38.1 (± 2.3) cycles]. DFA had poor sensitivity, missing 17 samples that were positive by both culture and the *IS481 *HP assay, as well as 28 samples that were positive by the *IS481 *HP assay, but negative by culture. Six specimens that were DFA positive, tested negative by both culture and PCR and are most likely false positives due to the subjective nature of DFA interpretation. Seven specimens were overgrown in culture, 4 of which were negative by PCR and DFA and 3 were positive by PCR (1 of these 3 was positive by DFA).

### Comparison of real-time PCR methods for the detection of *B. pertussis *and *B. holmesii*

The analytical sensitivity of the *IS481 *MB assay was determined using a serial dilution of purified DNA of *B. pertussis *ATCC 9797 with a detection limit of 10 cfu/ml (1 cfu input) which is similar to that reported previously [8]. Thus a 10-fold difference in end point sensitivity was noted when compared with the *IS481 *HP assay. The *IS481 *MB assay gave average Ct values 4–5 cycles higher for positive patient specimens than the *IS481 *HP assay (Table 1).

**Figure 4:**
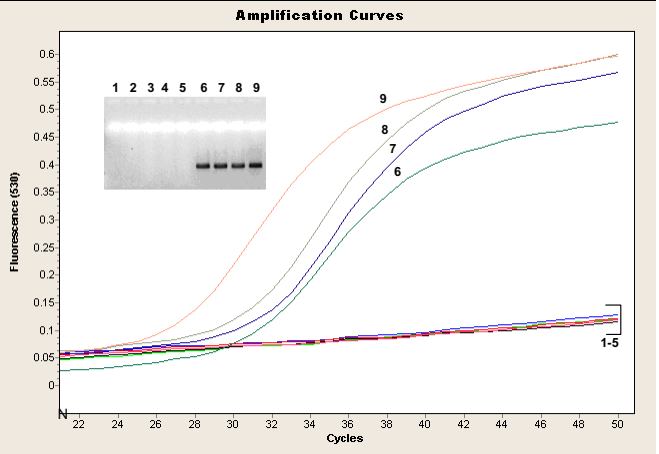
**LightCycler™ amplification profile of the *IS481 *MB assay with *Bordetella *isolates **The samples correspond to: 1 = *B. bronchiseptica *ATCC 10580, 2 = *B. bronchiseptica *ATCC 29513, 3 = *B. parapertussis *ATCC 1533, 4 = *B. parapertussis *33047, 5 = *B. parapertussis *33205, 6 = *B. holmesii *ATCC 51541, 7 = *B. holmesii *98P1995-F, 8 = *B. holmesii *98P0971-D, 9 = *B. pertussis *ATCC 9797. The specific amplified product is 154 bp and the agarose gel of products is given as an inlay (numbers correspond to samples in the amplification curves). Stocks of *Bordetella *isolates were approximately 10^6^cfu/ml unless otherwise quoted. MB = molecular beacon

**Figure 5:**
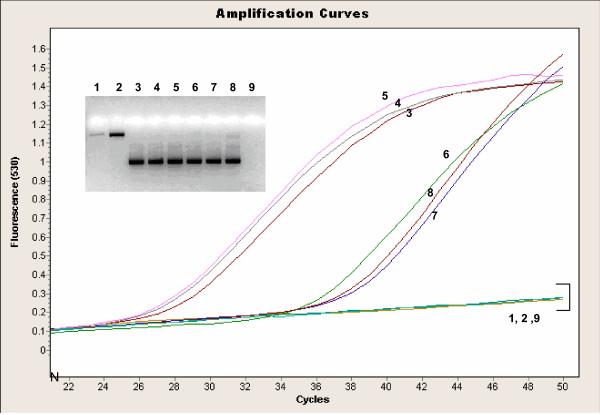
**LightCycler™ amplification profile of the *IS1001 *MB assay with *Bordetella *isolates **The samples correspond to: 1 = *B. bronchiseptica *ATCC 10580, 2 = *B. bronchiseptica *ATCC 29513, 3 = *B. parapertussis *ATCC 1533, 4 = *B. parapertussis *33047, 5 = *B. parapertussis *33205, 6 = *B. holmesii *ATCC 51541, 7 = *B. holmesii *98P1995-F, 8 = *B. holmesii *98P0971-D, 9 = *B. pertussis *ATCC 9797. The specific amplified product is 186 bp and the agarose gel of products is given as an inlay (numbers correspond to samples in the amplification curves). MB = molecular beacon.

The *IS481 *MB and *IS1001 *MB assays [8] utilize primers and probes that amplify and detect a 154 bp fragment in the *IS481 *region specific to *B. pertussis *and *B. holmesii *(Figure 4) and a 186 bp region in the *IS1001 *region, specific to *B. parapertussis *and *B. holmesii *(Figure 5), respectively. Combined results from both reactions can resolve whether a specimen is positive for *B. pertussis, B. parapertussis *or *B. holmesii*. In our experiments, the *IS1001 *primers gave a non-specific product of approximately 700 bp for two *B. bronchiseptica *strains, but this product was not recognized by the MB probe (Figure 5). Further assessment of specificity for this assay was not undertaken during this study but has been evaluated elsewhere [8].

Several previously extracted patient samples were retested by the *IS481 *HP assay to ensure the integrity of the extracted DNA over time (data not shown). Eleven positive specimens with an average Ct value of 25.2 (± 4.8) cycles on the original PCR run, remained positive with an average Ct value of 25.9 (± 5.1). This difference was considered insignificant, indicating that the DNA in these specimens had not degraded over time, and that subsequent retesting with the *IS481 *MB and *IS1001 *MB assays for comparison was valid.

All 66 samples positive by the *IS481 *HP assay were retested by both the *IS481 *MB and *IS1001 *MB assays. Table 1 compares these 66 specimens in relation to PCR and conventional results, including average Ct values (± SD). Sixty-four of 66 (93.0%) of these samples confirmed positive for *B. pertussis *by the *IS481 *MB assay. The 2 samples that did not confirm were weak positives as they were also missed by both DFA and culture and had high Ct values on the *IS481 *HP assay (39.7and 39.1 cycles). No *B. holmesii *was detected in this population by the *IS1001 *MB assay. A random selection of 29 samples that were negative by the *IS481 *HP assay were also screened by the *IS481 *and IS1001 MB assays. No further positives were obtained by the *IS481 *MB assay but 2 specimens that were culture positive for *B. parapertussis *were detected by the *IS1001 *MB assay, as expected.

### TPR hydrolysis probe assay

**Figure 6:**
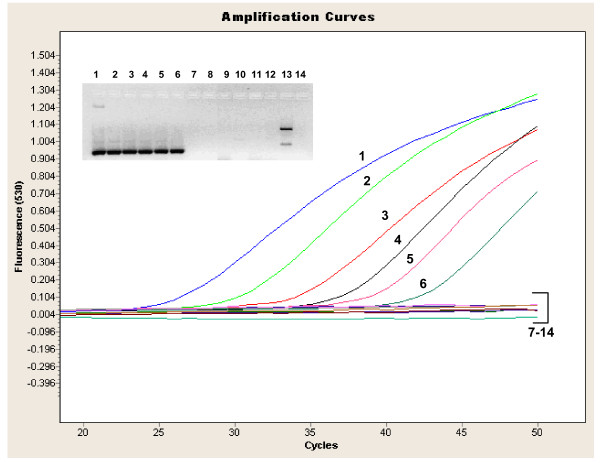
**LightCycler™ amplification profile of the TPR assay with *Bordetella *isolates. **The samples correspond to: 1–8 = dilution series of *B. pertussis *ATCC 9797 (10^8^cfu/ml to 10^1^cfu/ml, respectively), 9 = *B. bronchiseptica *ATCC 10580, 10 = *B. bronchiseptica *ATCC 29513, 11 = *B. parapertussis *ATCC 1533, 12 = *B. parapertussis *33047, 13 = *B. holmesii *ATCC 51541, 14 = *B. holmseii *98P1995. The amplified product is 167 bp and the agarose gel of products is given as an inlay (numbers corresponding to samples in the amplification curves). Stocks of *Bordetella *isolates were approximately 10^6^cfu/ml unless otherwise quoted.

This novel real-time PCR assay showed high specificity for *B. pertussis *and did not pick up cultured *B. holmesii*, *B. bronchiseptica *or *B. parapertussis *(Figure 6). Analytical sensitivity of this assay was performed on a serial dilution of purified DNA of *B. pertussis *ATCC 9797 and detected 100 cfu/ml, a 10–100 -fold difference from the *IS481 *HP and *IS481 *MB assays. No difference in limit of detection was noted whether analysis of amplified products was by agarose gel electrophoresis or using the TPR probe. Thus the difference in sensitivity when comparing this assay to those targeting insertion sequences was more likely related to the difference in template copy number than design of the probe. The conventional assays targeting TPR (used in the developmental stages of the final real-time assay) gave the same limit of detection when analysed by agarose gel electophoresis as products from the LightCycler™ assay (1000 cfu/ml stock concentration or 10 cfu input into the assay). Interestingly, one of the *B. holmesii *isolates gave two higher molecular weight bands than the expected specific product when analyzed by agarose gel electrophoresis (lane 13 from gel inlay of Figure 6). These products were not picked up by the specific probe, however.

All 66 samples positive by the *IS481 *HP assay were retested by the TPR assay (Table 1). The TPR assay was able to detect 100% of the specimens where the *IS481 *HP assay, *IS481 *MB assay, culture and DFA were all positive. The sensitivity of this assay decreased slightly where DFA was unable to detect positives. Sensitivity was 50% for specimens which were culture negative but *IS481 *HP assay positive.

Patient data collected after this study (June to October 2004) remained consistent with the results presented above (data not shown). *B. pertussis *was detected in approximately 10% of the specimens and *B. holmesii *was absent (using the *IS1001 *MB assay). Of the 16 positive specimens detected by the *IS481 *HP assay (from a total of 131 tested), all were detected by the *IS481 *MB assay, and 12 were detected by the TPR assay. The 4 positives that the TPR assay missed were weak positives with high Ct values on the *IS481 *HP and *IS481 *MB assays.

## Discussion

PCR has been shown to be more sensitive than culture, resulting in culture negative/PCR positive specimens [2,9-14]. Only a few studies have reported cases of culture positive/PCR negative samples and most of these were earlier papers with interpretation of results complicated by the following factors: unequal sample splitting, lack of DNA extraction procedures, inhibited PCR reactions and/or lack of sensitive product detection methods.

Real-time PCR assays with *IS481 *have been published [6-8,15-18]. Advantages offered by real-time PCR include; elimination of post-amplification handling thereby reducing the risk of contamination, faster turn-around time and higher sensitivity [8]. We were able to confirm in this study the diagnostic utility of the previously published methods, adapt alternative assays targeting insertion sequences to rapid LightCycler™ format for confirmation of positive results, as well as design and develop an assay based on TPR for *B. pertussis*. In our study, the *IS481 *HP assay increased the yield of *B. pertussis *positive nasopharyngeal swabs by 1.7 fold (detecting 28 positives in addition to the 38 detected with culture). None of the specimens were culture positive/PCR negative. The *IS481 *HP and *IS481 *MB assays had analytical sensitivities of 1–10 cfu/ml (0.1–1 cfu input), which is consistent with previously published data [8].

A problem that we encountered with the *IS481 *HP assay was weak positives with high crossing thresholds ("high cycle positives") that were irreproducible with a second run of the same *IS481 *HP assay and/or with the *IS481 *MB assay. These were reported as not containing detectable pertussis DNA. It is unknown whether this type of result represents very low levels of *B. pertussis *in the specimen or a false positive signal.

One concern is that culture negative/PCR positive specimens may represent false positive PCR reactions rather than increased PCR sensitivity. As a first check for possible false positive reactions, we utilized assays targeting different regions of *IS481 *(the *IS481 *HP and *IS481 *MB assays) with good concordance for positive results. A further approach we used to help interpret *IS481 *positive results was to perform PCR against a different target gene (toxin promoter). If both gene target assays (against *IS481 *and toxin promoter) are positive, then it is reasonable to conclude that the specimen is truly *B. pertussis *positive. We developed a novel real-time PCR targeting the TPR for use as a second target to *IS481*. Currently there is no sequence available in GenBank for the TPR of *B. holmesii*, which presented a challenge in developing this assay. However, due to the sequence heterogeneity among other *Bordetella *species, we were able to design a highly *B. pertussis*-specific assay. We tested the specificity of the toxin promoter primers with isolates of *B. parapertussis*, *B. bronchiseptica *and *B. holmesii*. Even large amounts of DNA from these organisms failed to give false positive results using this assay. The TPR PCR had an analytical sensitivity of 1000 cfu/ml (10 cfu input) and is thus less sensitive than the assays targeting the *IS481 *region. This is in agreement with previous data [6] and is explained by the gene copy number of 50–100 for *IS481 *compared to 1 for the toxin promoter. We recognized that sensitivity would be a limiting factor of the toxin promoter target. Among the 66 specimens that were positive by the *IS481 *HP assay, there were 51 that also gave positive results with the TPR PCR. Of the 15 that did not confirm, 2 were culture positive and therefore represent false negatives with the TPR assay, which could possibly be due its poorer sensitivity or due to a polymorphism of the toxin promoter [19]. Thus, even with its 100 fold-lower analytical sensitivity, the TPR PCR was useful for confirming the majority of specimens.

The *IS481 *target for *B. pertussis *diagnosis has the limitation of also being present in *B. holmesii *[7,13,20] and some *B. bronchiseptica *strains (ATCC 4617, ATCC 14455) [9]. The *IS481 *sequence of *B. holmesii *and *B. pertussis *differs by only 2 bases [7]. One approach to decipher whether a specimen positive for *IS481 *is *B. pertussis *versus *B. holmesii *is to perform the *IS1001 *MB assay [8]. *B holmesii *contains the *IS1001 *but *B. pertussis *does not. *B holmesii *was not detected in any of our NP specimens, which suggests that it may not be unnecessary to rule out *B. holmesii *for specimens that are positive for *IS48*1 in the Alberta population. Our study did not include *B. bronchiseptica *ATCC 4617 or ATCC 14455 but this warrants further study. Interestingly, the *IS1001 *primers gave a product for *B. bronchiseptica *ATCC 29513 and ATCC 10580, which has not been previously reported. This product was not recognized by the MB probe, demonstrating the specificity provided by the probe.

Although PCR is a useful tool for *B. pertussis *diagnosis, it is still important to obtain isolated *B. pertussis *organisms for epidemiologic characterization. We found that both culture and PCR could be undertaken from a single NP swab. Elution of the swab in casamino acid allowed equal splitting of the specimen for DFA or culture and PCR, thereby removing sampling bias in the interpretation of results.

## Conclusion

In summary, the results of this study confirm that PCR is more sensitive than culture and DFA for diagnosis of *B. pertussis*. As a result of this work our laboratory has discontinued DFA and reports only *IS481 *HP PCR results to submitters. Specificity of reproducible positive specimens is 100% and *B. holmesii *is not prevalent in our population. We have been able to show that PCR is a suitable and practical approach to *B. pertussis *detection and diagnosis, providing rapid results, which are useful for management of individuals and potential outbreaks. We have explored use of additional tests in order to differentiate *B. pertussis *from closely related organisms, which may present with similar symptoms. Currently, all NP swabs are tested using the *IS481 *HP assay, and positive specimens are cultured. The *IS481 *MB assay is employed for specimens that are difficult to resolve using the *IS481 *HP assay and as a second line test. Retrospectively, patient samples are batched and tested by the *IS1001 *MB and TPR assays for research purposes and to ensure there are no changes in *B. holmesii *incidence in the population. The screening PCR assay is more sensitive than the other procedures, and its specificity is acceptable. Culture is maintained for PCR-positive specimens only, for epidemiologic purposes. Our follow up procedures confirm the validity of the front-line PCR test for *B. pertussis *in our laboratory.

## Competing interests

The author(s) declare that they have no competing interests.

## Authors' contributions

LK undertook the experiments for this study and prepared the draft methods and results sections of the manuscript. JA-B evaluated sample collection and storage and drafted the introduction and discussion of results for the manuscript. PT and JF were equally involved in the conception and design of the study as well as critical appraisal and revision of the manuscript. All authors read and approved the final manuscript.

## Pre-publication history

The pre-publication history for this paper can be accessed here:


